# Circular Dichroism of Gold Bipyramid Dimers

**DOI:** 10.1021/acs.jpclett.1c00792

**Published:** 2021-05-27

**Authors:** Radosław Deska, Patryk Obstarczyk, Katarzyna Matczyszyn, Joanna Olesiak-Bańska

**Affiliations:** Advanced Materials Modelling and Engineering Group, Wroclaw University of Science and Technology, 50-370 Wrocław, Poland

## Abstract

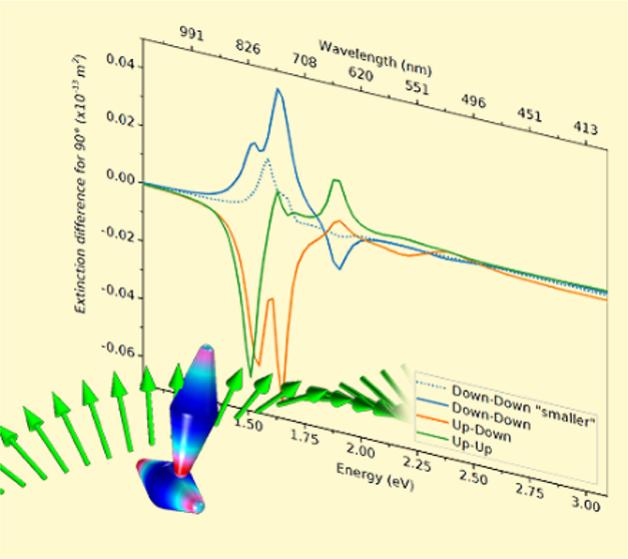

Chiral nanomaterials attract broad attention, as they offer new possibilities of modulation of optical properties and dissymmetry factors outperforming organic materials. Among various nanoparticles, plasmonic bipyramids present numerous advantages as building blocks of chiral nanomaterials (well-defined modulation of optical properties with the morphology of nanoparticles, narrow optical resonances, and high size and shape uniformity of synthesized particles). We study different possible orientations of gold bipyramids with respect to each other in dimers obtained by wet chemistry methods. For circularly polarized incident light we evaluate linear optical cross sections and plasmonic local field enhancement using COMSOL Multiphysics. We observe coupling of the nanoparticles’ local fields and thus changes in extinction spectra, which modulate chiroptical properties of dimers. To assess the chirality of various arrangements, we note differences in cross sections for left- and right-handed polarized light which we further evaluate as the dissymmetry *g*-factor. Our results provide BPs configurations with dissymmetry factor as high as −0.3.

Plasmonic nanoparticles (NPs) present unique optical properties originating from the surface plasmon resonance. Elongated plasmonic NPs, such as nanorods (NRs), exhibit transverse and longitudinal plasmonic modes (t-SPR and l-SPR, respectively), where the latter brings large local field enhancement and is very sensitive to the polarization of incident light and refractive index of the surrounding medium.^1,2^ Gold bipyramids (BPs) present even larger local field enhancement, larger optical cross sections,^3^ narrower resonances,^4,5^ high shape uniformity,^6^ and higher sensitivity to refractive index changes than NRs. Thus, they appear as promising candidates for various applications in biophotonics and spectroscopy.^7^

In the past decade, plasmon coupling in various relative orientations of NRs was explained^8−12^ based on plasmon hybridization theory,^13,14^ and circular dichroism of systems with two or more repeating NRs was shown to reach huge values of dissymmetry factor.^9−12^ Therefore, it is of great importance to understand how aggregates of bipyramids interact when excited with circularly polarized light and how plasmon coupling contributes to chirality in such systems. Few works describing chiral BP assemblies point at the higher CD signal obtained with BPs in comparison to NRs.^15^ However, to fully take advantage of BPs’ geometry, broader studies on the origin of chirality in these structures are needed.

In this work we show for the first time a systematic investigation of chiroptical properties of gold bipyramid dimers. We calculate optical cross sections for different alignments of gold bipyramids illuminated with circularly polarized light and analyze near-field plasmon coupling based on the plasmon hybridization theory. Individual NPs and their aggregates can be observed by means of optical imaging techniques like luminescence or dark-field microscopy. To retrieve the scattering spectrum of NPs one should refer to dark-field measurements with illumination in total internal reflection mode (which allows us to avoid external polarization artifacts).^11^ In the luminescence microscopy, gold nanoparticle excitation can be triggered either directly at the resonant frequency of a given plasmon or indirectly through interband transitions greater than 2 eV.^16^ To recover the full emission spectrum of a nanoparticle, one can refer to the latter excitation pathway only, even if it leads to inefficient emission and to broadening of luminescence bands due to increased damping in the interband transitions.^17^ This phenomenon enforces one to use significant incident power and/or long exposure times to overcome the low efficiency of emission.^18^ Our findings help us to apply both imaging methods mentioned earlier to determine dimer configurations for given (nearly) monodisperse nanobipyramids.

We performed a range of simulations with a commercial finite-element method (FEM) software, COMSOL Multiphysics, where the optical properties of BP dimers were determined. To work with experimentally relevant systems, we synthesized two sizes of BPs according to the procedure described in the Methods section in the Supporting Information, and we measured extinction spectra (Figure S1). Based on TEM images, the size of the nanoparticles was set to 102 × 36 nm and to 68 × 24 nm, for the long and short axes, respectively. From now on, we will refer to BPs of these sizes as larger and smaller BPs, respectively. The dimerization procedure was applied to these BPs, and the resulting aggregates were observed under TEM (see Figures S2 and S3). Then, we built a series of configurations which reflect experimentally observed dimers. We chose a range of *d*, θ, and Up–Up, Up–Down, and Down–Down configurations to perform full characterization of optical properties of dimers, as described in detail in the Methods section in the Supporting Information.

To ensure that chirality simulations are not affected by substrate, we simulated reference single bipyramid absorption resonance spectra excited with a circularly polarized electromagnetic plane wave. From absorption spectra on a substrate (Figure S4, 1–2b) we find that the resonant energy of longitudinal surface plasmon resonance (l-SPR) for larger BPs on the substrate is 1.597 eV with a full-width at half-maximum (fwhm) of 0.103 eV and that the numerical noise due to meshing observed as a nonzero *g*-factor across the simulated spectrum of an achiral particle on the substrate is negligible both for larger and smaller BPs. Similar results for BPs in water indicate that indeed no additional chirality is introduced by the substrate (see Figure S4, 1–2a). Having acknowledged that, we can proceed to the results of the investigation of the chirality of BPs.

## Absolute Differences

**Figure 1:**
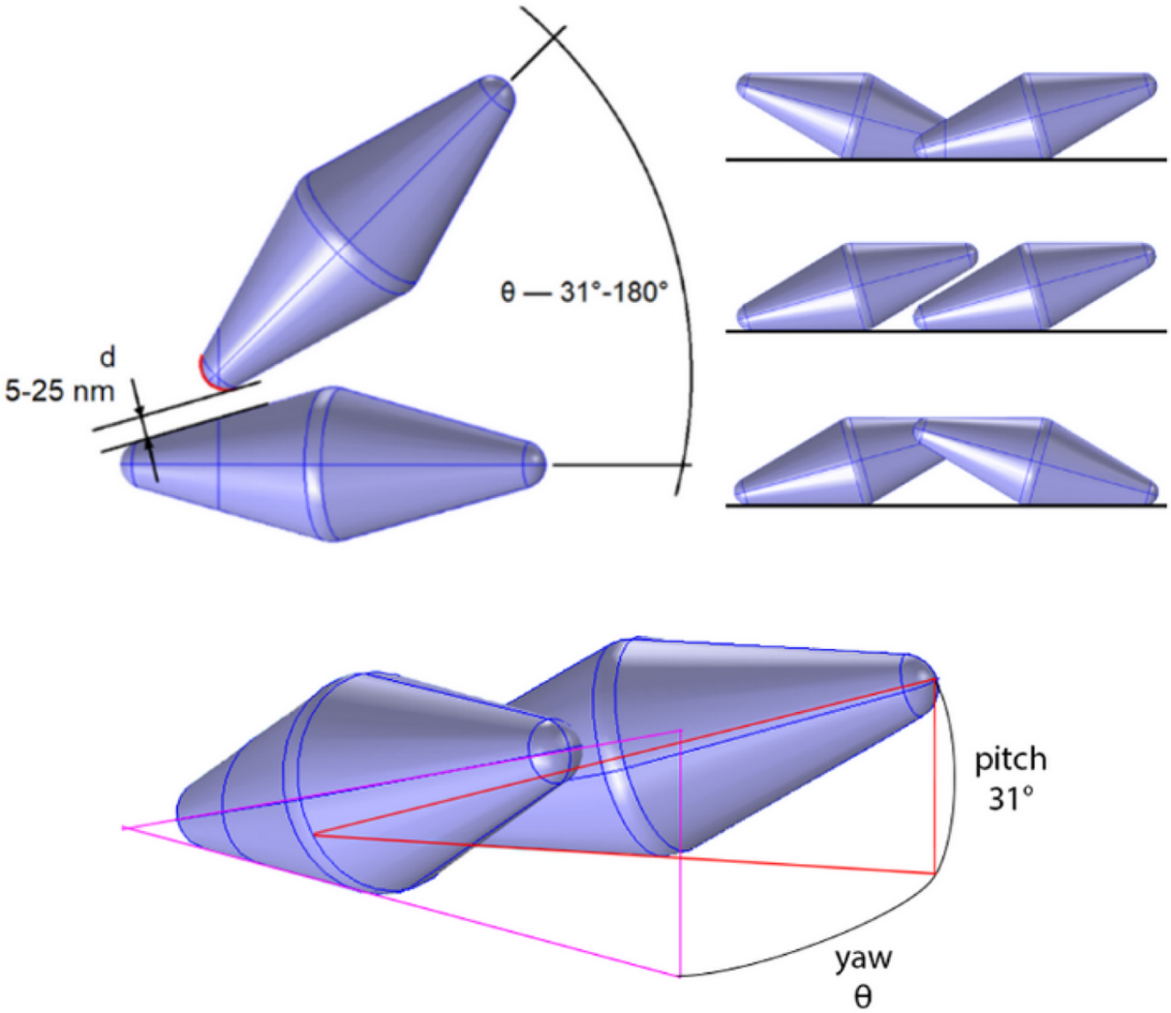
Simulated geometry of bipyramid dimers. Top view and oblique projection showing specific angles and distances on the example of Down–Down configuration. In the top-right corner front view of simulated configurations (from top to bottom): Down–Down, Up–Down, and Up–Up. Detailed description in the text.

**Figure 2:**
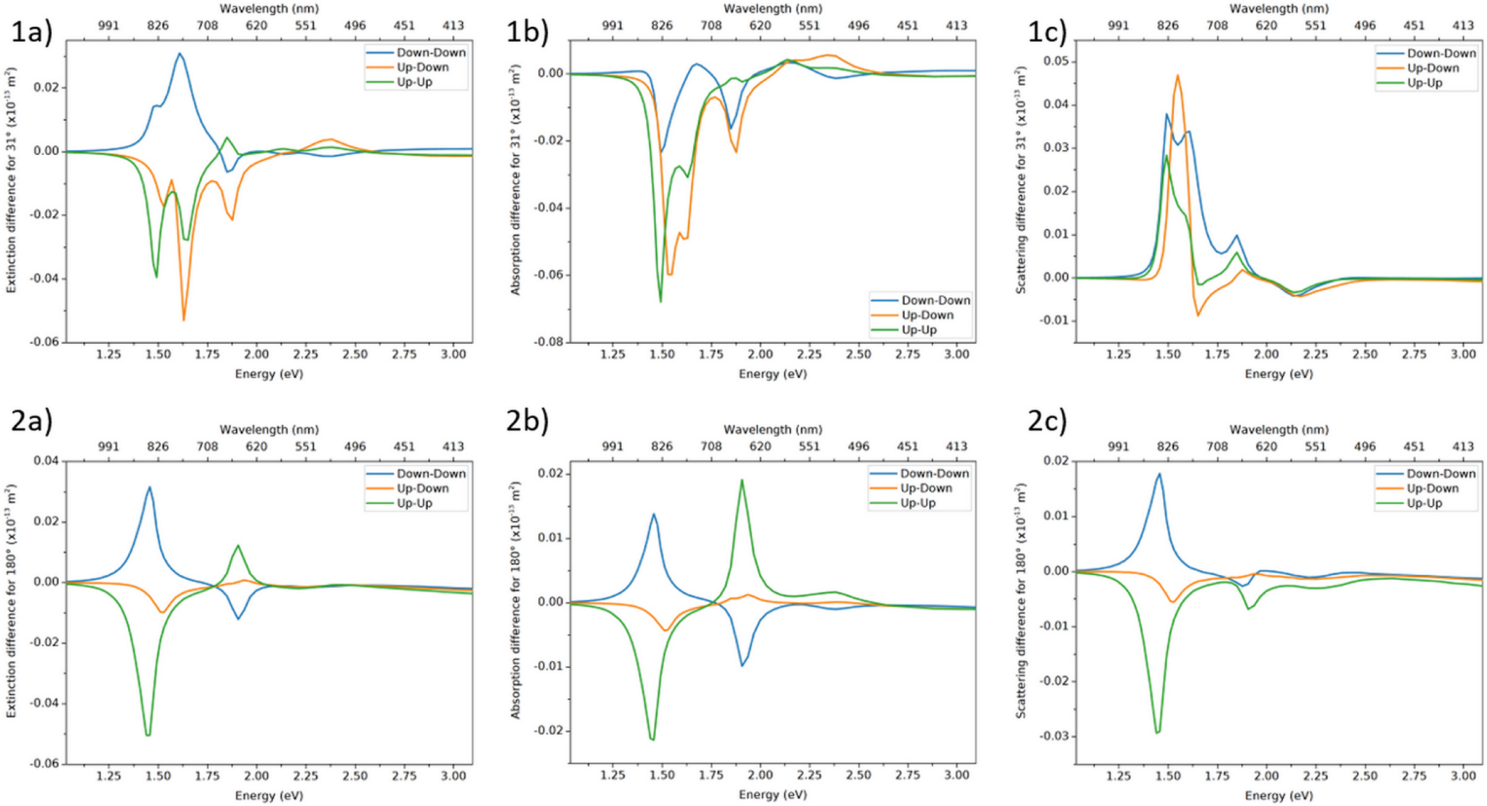
Extinction (1–2a), absorption (1–2b), and scattering (1–2c) absolute difference between left- and right-handed circularly polarized light for larger BP dimers characterized by yaw angles of 31° (1a–c) and 180° (2a–c).

. We considered bipyramid dimers lying on a glass surface with specific parameters of the system described in the Methods section. The probable mutual positioning of BPs is denoted as Up–Up, Up–Down, and Down–Down configurations (see Figure 1). In Figure 2 we present the spectra of the absolute difference in extinction (ADE), absorption (ADA), and scattering (ADS) of left- and right-handed circularly polarized light illuminating each of these configurations for two limiting cases of yaw angles of 31° and 180° (the optical cross sections, absolute differences, and *g*-factors spectra for all combinations can be found in Figures S5–S7). There are several characteristics to point out therefrom. (I) The most striking feature of the absolute difference spectra is extinction sign reversal between Down–Down and Up–Up configurations (Figure 2, 1–2a) across all yaw angles (cf., Figure S5, 2a–e). The reversal is not complete, though, most likely due to different refractive indices in the contact area between BPs in Down–Down versus Up–Up configuration.^19^ (II) Another marked feature is sign reversal between ADA and ADS for the same configurations in the most closed structures (31°) (i.e., Figure 2, 1b–c), which in effect decreases the ADE, especially for hybrid modes below 1.8 eV procured by coupling of l-SPRs. The extinction canceling-out is not complete for either of the configurations, which holds for angles below 90°; for and above angles of 90°, absorption and scattering differences yield the same sign (Figures S5–S7, 2a–e), thus leading to summing of the differences in the ADE spectrum. If we look closer at ADS spectra, the Down–Down configuration does not show sign reversal and stays positive at any angle. On the other hand, Up–Up and Up–Down configurations display sign reversal from positive to negative as the angle changes from 31° to 180° (Up–Down configuration gets nearly achiral at 180°). In the case of ADA spectra, the situation is reversed; Up–Up and Up–Down configurations do not exhibit sign reversal and stay negative at any angle (here, again, the Up–Down configuration is nearly achiral at 180°). Down–Down configuration presents sign reversal from negative to positive as the angle changes from 31° to 180°. Reassuming, for some yaw angles in any of the configurations, one should expect different results of chirality from absorption- and scattering-based measurements.

## Relative Differences

**Figure 3:**
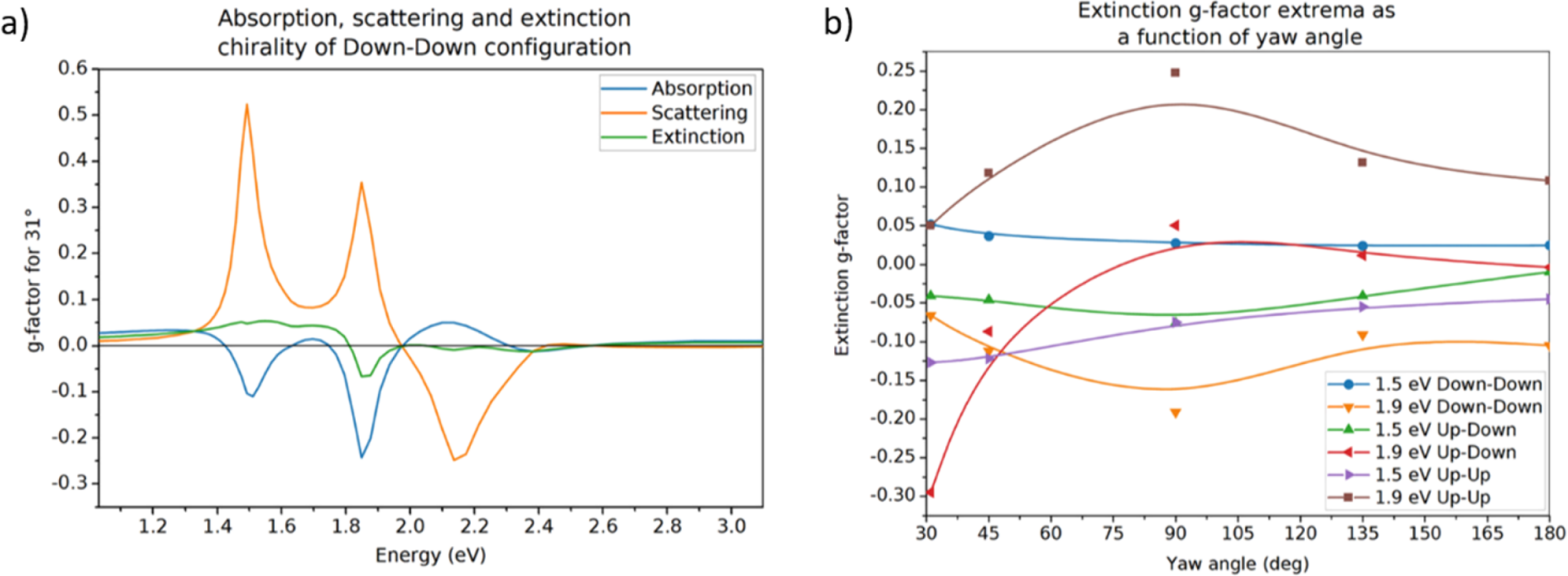
(a) Absorption, scattering, and extinction *g*-factor for the Down–Down configuration at 31° yaw showing absorptive contribution to sign of extinction chirality. (b) Extinction *g*-factor extrema at ∼1.5 eV and ∼1.9 eV hybrid resonances as a function of yaw angle between larger BPs. Solid splines were added to guide the eye.

. Usually, when comparing effectiveness of chirality in various optically active structures, one refers to the *g*-factor (eq 1 in the Methods section in the Supporting Information) which measures the absolute difference relative to an average spectrum, thus being insensitive to sample concentration. Let us then consider absorption, scattering, and extinction *g*-factor spectra named RDA, RDS, and RDE, respectively. From analysis of extinction *g*-factors (Figure S5, 3a–e) one can see that for all angles the Down–Down and Up–Up configurations show nearly mirrored magnitude of chirality for energies above 1.8 eV (which fall on higher-order resonant modes). This is mainly related to the absorptive part of extinction (see Figure 3a and Figure S6), in which this mirroring is present with similar line shape. For energies below 1.8 eV this mirror-like symmetry is lost. The largest absolute differences are observed for the hybrid plasmon modes at ∼1.5 eV and ∼1.65 eV, which result from coupling of l-SPR modes at the two BPs (we use a tilde to generalize on the plasmon resonance energy at any considered configuration or yaw between BPs). So, in other words, only the coupling of the first two hybrid modes is strong enough to enhance small changes between left- and right-handed polarized light extinction. However, the relative change is largest in the case of the smallest absolute absorption cross-section difference falling on hybrid plasmonic mode at ∼1.9 eV, for which the chirality is most pronounced across all angles, with the highest-valued results yielded for 90° (Down–Down and Up–Up configurations, reversed signs) and for 31° (Up–Down configuration). To better visualize this result, we plotted the extinction *g*-factor versus yaw angle (Figure 3b) in which we present the values at the resonances for which there is any symmetry in trends across yaw angle for different configurations. We observe the largest extinction in the case of the Up–Down configuration at 31° yaw with a *g*-factor value of −0.3 and also high values of −0.20 (Down–Down configuration) and +0.25 (Up–Up configuration) at 90° yaw. The values are comparable with the best top-down and bottom-up nanostructures^9−12,20,21^ in the visible range of the spectrum. The *g*-factors of ∼1.5 eV resonance do not vary much with angle. They all seem nearly independent of angle for any of the three configurations. The biggest variations are observed at ∼1.9 eV hybrid mode, the chirality of which changes from a large negative to a small positive value, when sweeping from 31° to 180° in Up–Down configuration, and in the other two configurations it reaches a maximum at 90°.

**Figure 4:**
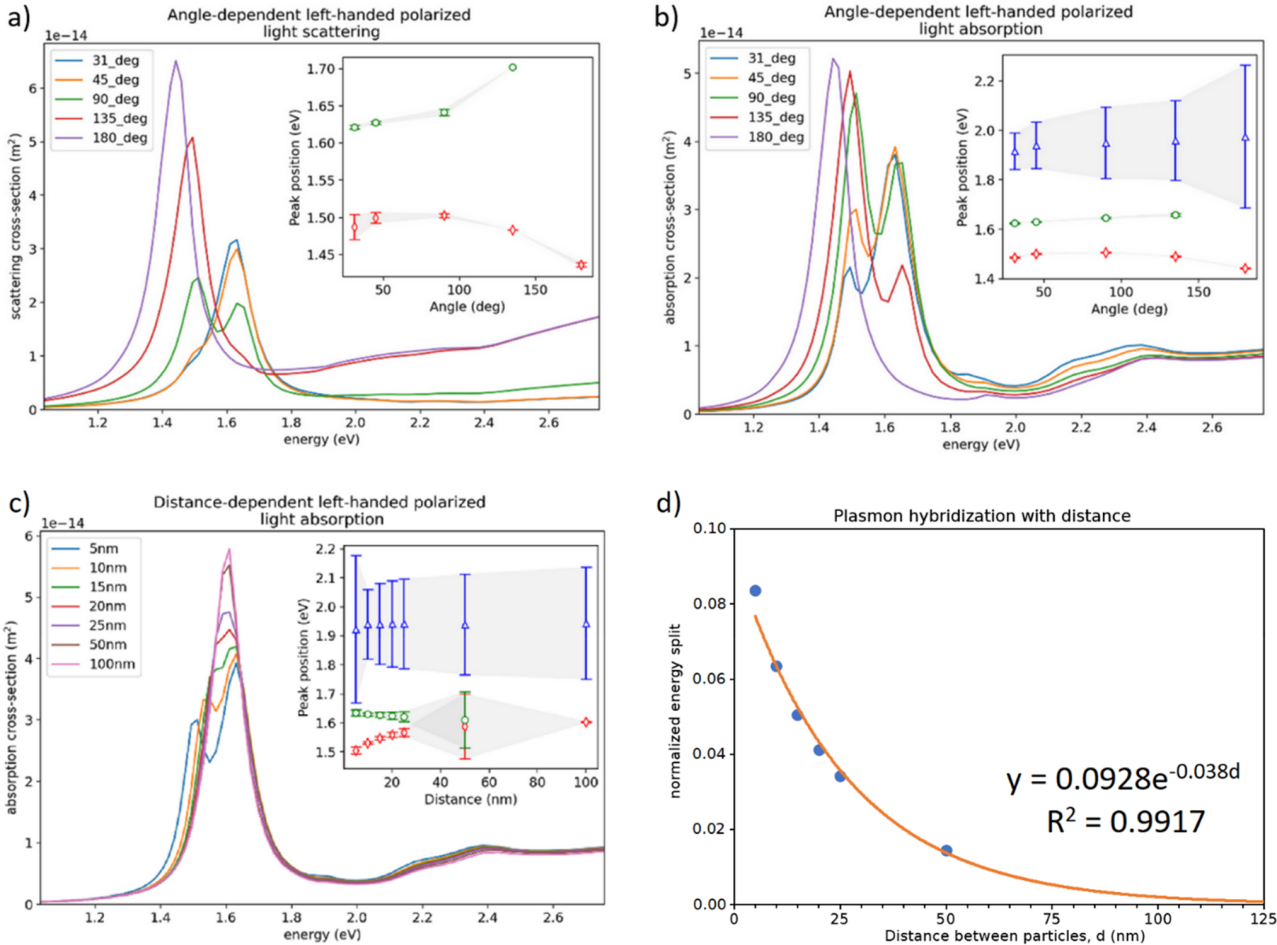
(a) Scattering and (b) absorption spectrum of left-hand polarized light changing with yaw angle, (c) absorption spectrum of left-hand polarized light, and (d) energy split changing with interparticle distance at θ = 45° for the Down–Down configuration for the larger BPs. Energy split is normalized by l-SPR energy in the single-particle limit and fitted with the plasmonic ruler equation (fitting parameters shown in the plot area). In the insets of a–c, red, green, and blue points refer to the three lowest energetic resonance peaks (as fitted with multiple Lorentzian fit). The insets show the peak positions shifting with the angle (a, b) and distance (c). Error bars indicate standard error of the fitted peak positions.

Let us delve into the source of the ∼1.9 eV hybrid mode. Until now, we assumed that ∼1.9 eV resonance is the result of coupling between BPs. However, if we look at single BP spectra of either a larger or smaller bipyramid, this assumption may no longer be valid as this resonance is also present in this single bipyramid case (see Figure S4). We made additional simulations for single BP to check for the source of ∼1.9 eV resonance. This simulation included three different linear polarization angles (0°, 45°, and 90° relative to long axis projection on the wavefront plane) and 0° and 31° pitch (as defined in Figure 1). The results of the absorption and scattering cross-section simulations presented in Figure S8 indicate that the ∼1.9 eV resonance is a property resulting from the nonzero excitation of transverse modes at nonzero pitch and its coupling with the strong longitudinal mode. Moreover, this resonance is absorptive in nature, being dark in the scattering mode (Figure 4a and Figure S8b). One could expect dependency of the peak position on the yaw between BPs; however, there is no such trend in the peak position (Figure 4b, inset). We conclude that ∼1.9 eV absorption resonance is excited by coupling of longitudinal and transverse modes. In the single BP at some nonzero pitch relative to excitation polarization this is by self-coupling of the modes (both being active in this case), whereas in the dimers this is additionally (as we consider BPs dimers with nonzero pitch) due to coupling of the l-SPR and proximity-induced t-SPR modes at the two nanoparticles.

Although this is not revealed by fitting of distance-dependent results for ∼1.9 eV due to large fitting error (inset in Figure 4c), similar coupling in dimers was observed for gold nanorods in T-configuration.^8^ Taking these considerations into account, we conclude that the hybrid mode at ∼1.9 eV exhibits the largest absorption difference at 90° for Down–Down and Up–Up configurations because in these situations the coupling between the l-SPR and t-SPR is the strongest across all yaw angles. The Up–Down configuration displays the strongest polarization for yaw angle 31°. This, however, needs further investigation.

## Determination of Dimer Configuration

. Once the single plasmonic pair spectra of absorption and/or scattering are obtained, the peaks and valleys of ADA and ADS spectra appear unambiguous and therefore useful for discerning a given configuration and angle. Therefore, by the analysis of the relative intensities of these peaks (see Figure S9) and valleys with a little help from the RDA and RDS spectra, one can give full information on geometry of such dimers, without the need of far-field emission or scattering pattern measurements by linearly polarized excitation sweeping. When describing absolute differences, we pointed out that Down–Down and Up–Up configurations are not fully mirror-symmetrical. That is good for estimation on whether the structure under investigation is left- or right-handed based on to-be-measured spectra. For structure estimation based on its scattering differential spectrum one has to take into account a detection threshold induced by ellipticities of applied optical elements.^11^ Thus, one needs to correct for this artifact, to consider measured chirality as an indicator of configuration and positioning of bipyramids in dimers. With a proper detection threshold the dimer structure may be estimated based on chirality spectra.

The above statements concerning the structure estimation refer to a very specific distance between the involved nanoparticles. Obviously, the distance between nanoparticles varies from pair to pair. Therefore, some differences are expected in the above results for other distances within dimers. The l-SPR mode hybridizes at short distances between two nanoparticles which appears as one resonance splitting into two with some energy difference. In Figure 4c the low-energy part of the split diminishes with increasing distance and shifts to the blue, while the high-energy part prevails and shifts to the red. The energy within the split decreases exponentially with the increasing distance, *d*, between nanoparticles (Figure 4d), which we confirmed by applying the plasmonic ruler equation.^22^ In the arbitrarily chosen spectral resolution limit of 2 meV the bipyramids are separated when at the distance of 112 nm, which agrees well with data reported previously.^8,23^

## Size-Dependent Chirality

. Finally, to see how chirality changes with nanobipyramid size, we considered the smaller version of BPs in the Down–Down configuration. The reduction of BP size was, in general, followed by reduction of chirality (Figures S5–S7). Although chirality of extinction diminishes within the whole simulated spectrum, it does not do so consistently for each resonance. Smaller BPs have slightly blue-shifted longitudinal resonance due to smaller electromagnetic phase retardation, and the van der Waals energy of interaction between two nanoparticles in a dimer strongly depends on the nanoparticle size and distance.^24^ Strangely enough, smaller bipyramid dimers exhibit absorption difference of opposite sign relative to larger bipyramid dimers below 90°. Different results for smaller BPs obviously lead to conclusions concerning dimers structure estimation and design of dimers with expected chirality; to avoid large discrepancies from data expected based on simulations, a narrow size distribution of the nanoparticles is required.

In this work we presented the remarkable chirality of gold nanobipyramid dimers. This chirality is reflected in huge values of *g*-factor, even −0.3 for Up–Down configuration at 31° yaw, coming from only two nanoparticles. In view of previous works showing increasing chirality with the number of particles,^25^ one can expect even stronger chirality of systems based on multiple gold nanobipyramids with possible applications in polarization or molecules sensing. The chiral response of the presented systems is affected by the interparticle angle and refractive index of the medium leading to changes in dichroism spectra as extreme as sign reversal for angles above a certain value. The refractive index leads to asymmetric chiral response when the configuration is reversed (Down–Down to Up–Up). The exact mechanism behind the size influence on chiral response requires further investigations. We also suggest that based on simple microscopic measurements one may determine the geometry of gold nanobipyramid dimers or design a specific chiral optical response of a nanostructure due to unambiguous differential optical spectra, given a narrow size distribution of the nanoparticles.

## Supporting Information Available

Detailed methods of simulation, synthesis methods, and TEM imaging description; full results of optical spectra for all combinations of dimers, as described in the methods section; additional heuristic simulations of single nanobipyramids optical spectra; and images which did not fit in the main text (PDF)
