# Supplementary material for: Circular Dichroism of Gold Bipyramid Dimers
Source: J Phys Chem Lett. 2021 May 27;12(21):5208–13. doi: 10.1021/acs.jpclett.1c00792 (PMC8279732; doi:10.1021/acs.jpclett.1c00792)
Supplement: Supplementary file 1 — jz1c00792_si_001.pdf [file jz1c00792_si_001.pdf]

Circular Dichroism of Gold Bipyramid Dimers

*Radosław Deska, Patryk Obstarczyk, Katarzyna Matczyszyn, Joanna Olesiak-Bańska**

Advanced Materials Modelling and Engineering Group, Wroclaw University of Science and Technology, Poland

SUPPORTING INFORMATION

1. BP geometry definition

A single particle geometry was built as two cones joint at the base and rounded at the tips and in the joining region ("bulk" in the table below). The initial dimensions are defined through final particle dimensions (which are known from TEM imaging) and rounded as described in the table below (in parentheses the values for the "smaller" bipyramids were given):

parameter name	expression	description
l0	102 (68) [nm]	nanoparticle final length
rtx	15 (10) [nm]	length loss after tip round
L	$\mathrm{l0} + 2 \cdot \mathrm{rtx}$	NP initial length
d0	36 (24) [nm]	NP final diameter
rbx	0.28 (0.1866) [nm]	diameter loss after tip round
D	$\mathrm{d0} + 2 \cdot \mathrm{rbx}$	NP initial diameter
rtip	$\frac{\mathrm{rtx}}{\sqrt{\frac{D^2 + L^2}{L}} - 1}$	tip roundness radius
rbulk	$\frac{\mathrm{rbx}}{\sqrt{\frac{D^2 + L^2}{L}} - 1}$	bulk roundness radius

2. Description of modelled physics

Using COMSOL Multiphysics 5.0.1.276, we solve Electromagnetic (EM) and Heat Transfer (HT) problem in Frequency-Stationary mode, that is the Maxwell equations are solved in the frequency domain and the HT equation is in the stationary form. We also assume no mass exchange in the model.

$$\nabla \times \mu_r^{-1}(\nabla \times \boldsymbol{E}) - k_0^2\left(\epsilon_r - \frac{j\sigma}{\omega\epsilon_0}\right)\boldsymbol{E} = 0$$

$$\nabla \cdot (-k\nabla T) = \frac{1}{2}\mathrm{Re}(\boldsymbol{J} \cdot \boldsymbol{E}^\star) + \frac{1}{2}\mathrm{Re}(i\omega\boldsymbol{B} \cdot \boldsymbol{H}^\star)$$

Total electric field, **E**, is the sum of incident (background) and scattered (relative) field. A general incident field is defined as a plane wave travelling in the $+z$ direction:

$$E_{b,x} = E_b \cdot \cos(\psi_0)\exp(-\mathrm{j} \cdot kz)\exp(\mathrm{j} \cdot 2\chi)$$

$$E_{b,y} = E_b \cdot \sin(\psi_0)\exp(-\mathrm{j} \cdot kz)$$

- linear polarization ($\chi = 0$ and $\psi_0 = [0, \pi)$)
- circular polarization
  - left-handed, *or counter-rotating as defined from the point of view of the source*:
    $$\psi_0 = \frac{\pi}{4}, \chi = -\frac{\pi}{4}$$
  - right-handed:
    $$\psi_0 = \frac{\pi}{4}, \chi = +\frac{\pi}{4}$$

The models were limited by Perfectly Matched Layer as a domain totally absorbing scattered field. The scattered wave was assumed to be a spherical wave sourcing from (0,0,0). Wave equations were solved separately for the nanoparticle(s), and the surrounding medium (also the substrate, if applicable).

In our model gradient of refractive index in surrounding water is considered. To solve in water domains, $\epsilon_r = n(\lambda, T)^2$ was taken as a function based on Bashkatov's and Genina's formula[1] containing information on both thermal and spectral dependence of water refractive index. The fitting parameters in this formula have been optimized to better reflect the data.[2-3]

$$n(\lambda, T) = A(T) + \frac{B(T)}{\lambda^2} + \frac{C(T)}{\lambda^4} + \frac{D(T)}{\lambda^6}$$

$$A(T) = 1.32151 - 1.02801 \cdot 10^{-5}T - 1.83882 \cdot 10^{-6}T^2 + 4.87625 \cdot 10^{-9}T^3$$

$$B(T) = 5244.54296 - 2.39947T - 0.044T^2 + 1.46191 \cdot 10^{-4}T^3$$

$$C(T) = -3.14031 \cdot 10^8 + 290367.7585T + 8.5412 \cdot 10^2T^2 + 1.10952T^3$$

$$D(T) = 5.18511 \cdot 10^{12} - 3.52388 \cdot 10^9T - 3.57241 \cdot 10^7T^2 - 4.31619 \cdot 10^4T^3$$

where λ is given in [nm] and T is given in [degC].

To solve in gold domains, complex relative permittivity values were taken from Johnson and Christy[4].

Heat capacity at constant pressure was taken from Sassaroli et al.[5] We simulate nanoparticles deposited on glass, with relative permittivity, thermal conductivity and heat capacity at constant pressure taken from Lide's CRC Handbook[2].

Electromagnetic wave absorption, or dissipation, comes from resistive heating and magnetic losses within a material, therefore absorption cross-section can be defined as ratio between integral of power loss density over the absorbing volume, Q_h, [W] and incident energy flux, I_{inc}, [W/m^2] (explicitly input as 1 [GW/m^2])[6]:

$$\sigma_{abs} = \frac{\int_V Q_h}{I_{inc}}$$

We calculate absorption cross-section in the nanoparticles' domains only, since the electromagnetic loss due to conductivity of gold is of many orders of magnitude larger than the losses in the surrounding media.

Scattering cross-section is defined as ratio between scattered power [W] to incident energy flux [W/m^2] (calculated as the integral of squared ratio between scattering amplitude norm, $\boldsymbol{E_{ff}}$, and background amplitude, E_b, referred to far-field transform sphere surface of radius r_{ff}):

$$\sigma_{sca} = r_{ff}^{-2} \cdot \boldsymbol{r_0^2} \cdot \int_S \left(\frac{\boldsymbol{E_{ff}}}{E_b}\right)^2 dS$$

3. Methods

We used commercial finite-element method (FEM) software, COMSOL Multiphysics 5.0.1.276, to perform simulations of gold BPs based on TEM images of synthetically prepared bipyramids (Figure S2 and Figure S3 in supplementary information). The bipyramids were modelled as bicones with tips and central part rounded. We calculated extinction spectra of the BP dimers for left-handed and right-handed circularly polarized light (LCP and RCP, respectively) with a distance, *d*, and the yaw angle, *θ*, between the constituent nanoparticles, see Figure 1 in the main text. The distance is defined in the projection plane perpendicular to incident beam as a gap between curved tip of one BP and the other BP side. The distance and the yaw angle vary in the range 5-25 nm and 31-180°, respectively, where the minimal angle 31° results from BP aspect ratio (AR), based on initial (non-rounded) geometry of BP (see Section 1 above). The angular dependence was solved for the distance of 5 nm. The distance dependence was solved for the angle of 45°. We consider the bipyramids lying on a glass surface with the long axis of each BP pitched at 31° to the surface (again, this angle results from the aspect ratio; the angle was not changed during our simulations of chirality), yielding adjacency of BP's one side to the surface to reflect most probable positionings of single bipyramid on surface (cf. Up-Up, Up-Down, Down-Down configurations by Sivun et al.[7]). From the computation solutions we evaluated: absorption cross-section (resistive and magnetic power loss divided by incident irradiance that was set at 1 GW/m^2), scattering cross-section (far-field radiation power normalized by the surface of far-field transform sphere and divided by incident irradiance), and extinction cross-section (the sum of absorption and scattering cross-sections). We then calculated a dissymmetry g-factor for each cross-section, which defines the unitless circular dichroism as the LCP (σ_{LCP}) and RCP (σ_{RCP}) cross-section difference normalized by

average cross-section of LCP and RCP, which allows for comparison of different optical systems. For details of modelled physics, see Section 2 above.

$$g(\lambda) = \frac{2\left(\sigma_{LCP}(\lambda) - \sigma_{RCP}(\lambda)\right)}{\sigma_{LCP}(\lambda) + \sigma_{RCP}(\lambda)} \qquad (1)$$

Nanoparticles synthesis and dimerization. Gold bipyramids (BPs) were synthesized following the procedure by Sánchez-Iglesias et al.[8], although we replaced citric acid with sodium citrate during seed preparation. **SEED**. Briefly, 14.71 mg of trisodium citrate was dispersed in 9.338 mL of ultrapure water (conductivity < 0.06 µS) and then 3.91 µL of 0.6347 M $HAuCl_4$ was added. Next, 661 µL of 25 wt % CTAC in water was added to the mixture under gentle stirring and 250 µL of 0.025 M $NaBH_4$. The color change (from yellow to brown) was observed. After 2 minutes of vigorous stirring, the vial was closed and solution was heated in oil bath at 80 °C for 90 minutes under stirring, leading to color change from brown to red. The seed solution was stored in room temperature. **BPs GROWTH**. 50 mL of 0.1 M CTAB was mixed with 2.5 mL of 0.01 M $HAuCl_4$ solution. Next, 0.5 mL of 0.01 M $AgNO_3$, 1 mL of 1 M HCl and 0.4 mL of 0.1 M ascorbic acid were added. Certain volume of gold seeds (0.1 – 2.5 mL) was added to the aqueous solution. The mixture was gently mixed and left in 30 °C for at least 2 hours. Then extinction spectra were measured. **DIMERISATION.** It was conducted following the experimental procedure by Malachosky and Guyot-Sionnest[9]. Briefly, 125 µL of gold nanoparticles solution was placed in a 4 mL cuvette and mixed with 2.725 mL of ultrapure water, 100 µL of 30 mM HCl solution (final volume 3 mL and pH 3). UV/Vis spectrum was measured instantly. Then, 50 µL of 6 mM L-cysteine solution was added and extinction spectra were collected in 10-minute intervals to observe the progress of BPs binding.

Transmission electron microscopy. 1.5 mL of the solution after dimerization was transferred into 2 mL Eppendorf tube and centrifuged (4000 RCF, 10 minutes, 3 times) to remove supernatant containing most of the non-bound L-cysteine and excess CTAB. Pellet was re-dispersed in 5 mL of ultrapure water for storage. Solution containing washed gold bipyramids, dimers and oligomers was diluted 100 times to achieve separation of nanoparticles before manual drop-casting (10 µL) onto TEM grids (Agar Scientific, S160). TEM imaging was performed on Hitachi H-800 system under 150 kV where various mono-, di- and oligomers were found separated.

4. Figures

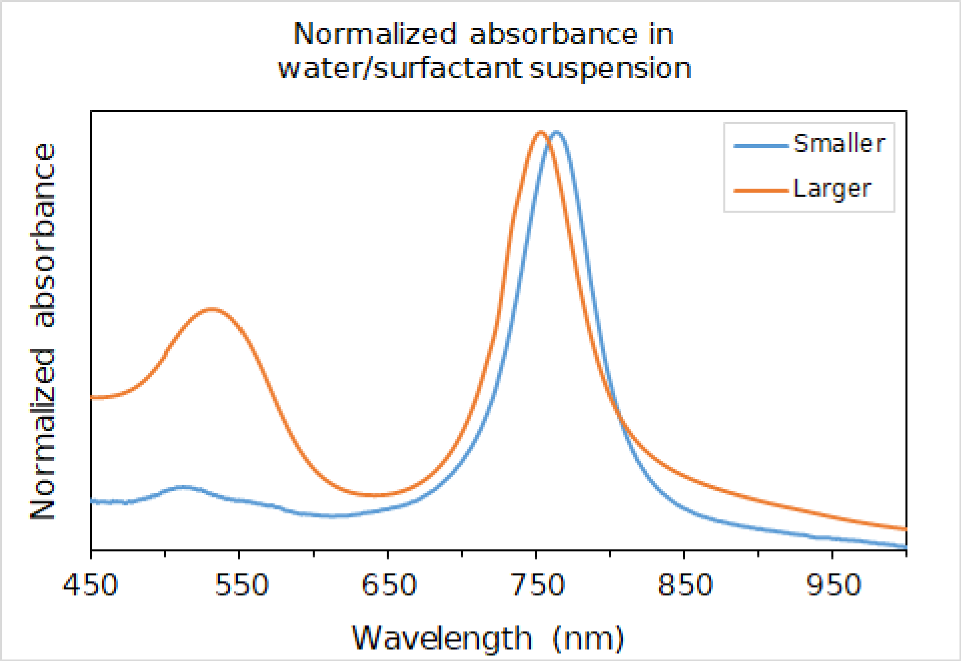

Figure S1 Absorbance spectra of nanobipyramids suspended in water/surfactant mixture. Labels “Larger” and “Smaller” in the legends refer to nanobipyramids in Figure S2 and Figure S3, respectively.

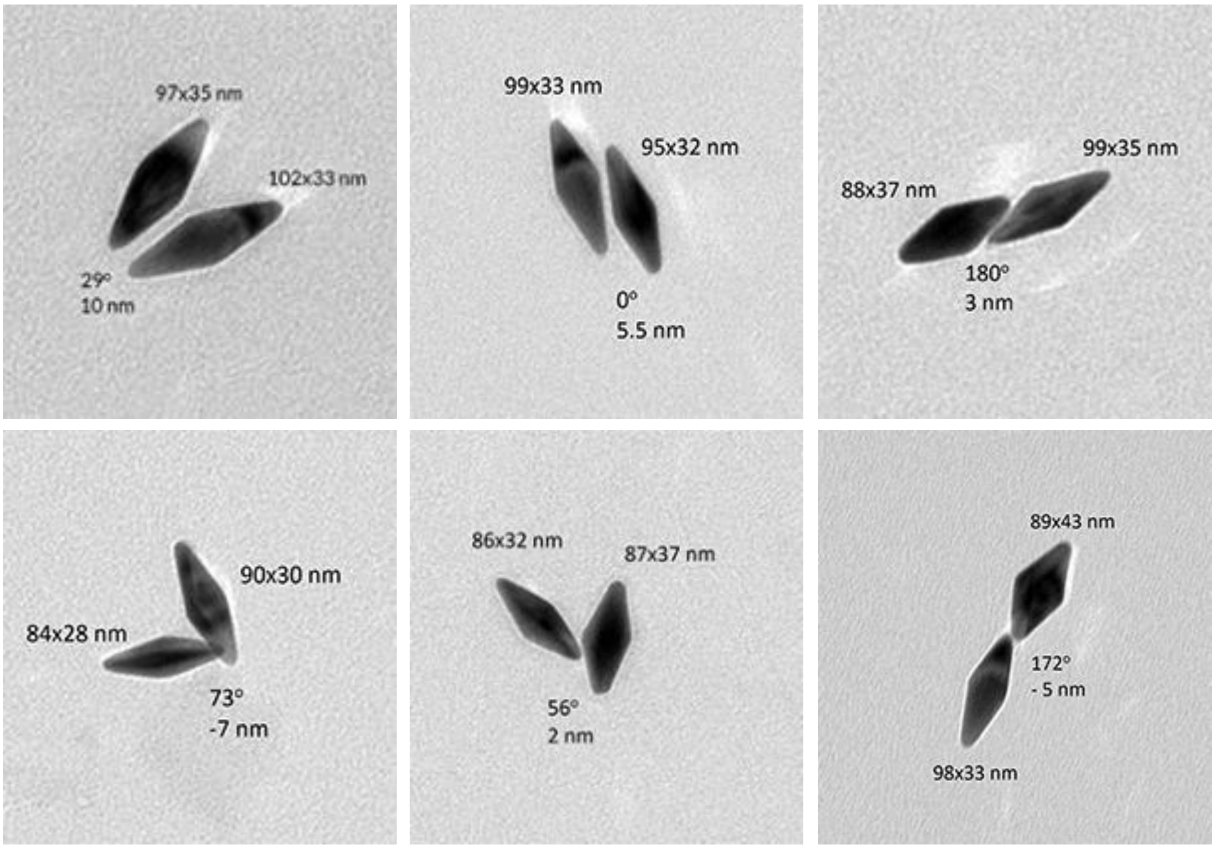

Figure S2 TEM images of nanobipyramids dimers – Sample with LSPR 751 nm. Average dimensions 93±7 × 35±4 nm. Indicated dimensions include long axis, short axis, non-reflex angle between long axes and distance (as defined in the main text).

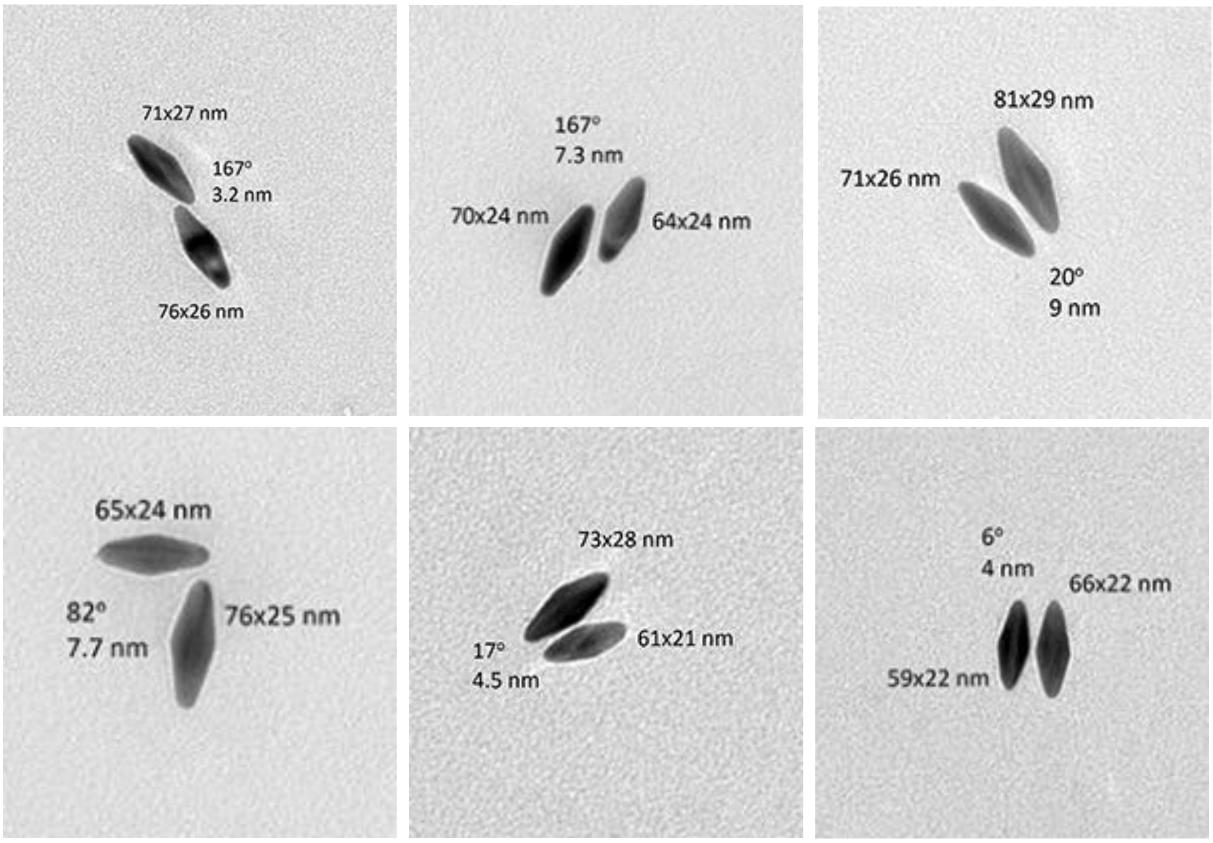

Figure S3 TEM images of nanobipyramids dimers – Sample with LSPR 764 nm. Average dimensions 71±6 × 27±3 nm. Indicated dimensions include long axis, short axis, non-reflex angle between long axes and distance (as defined in the main text).

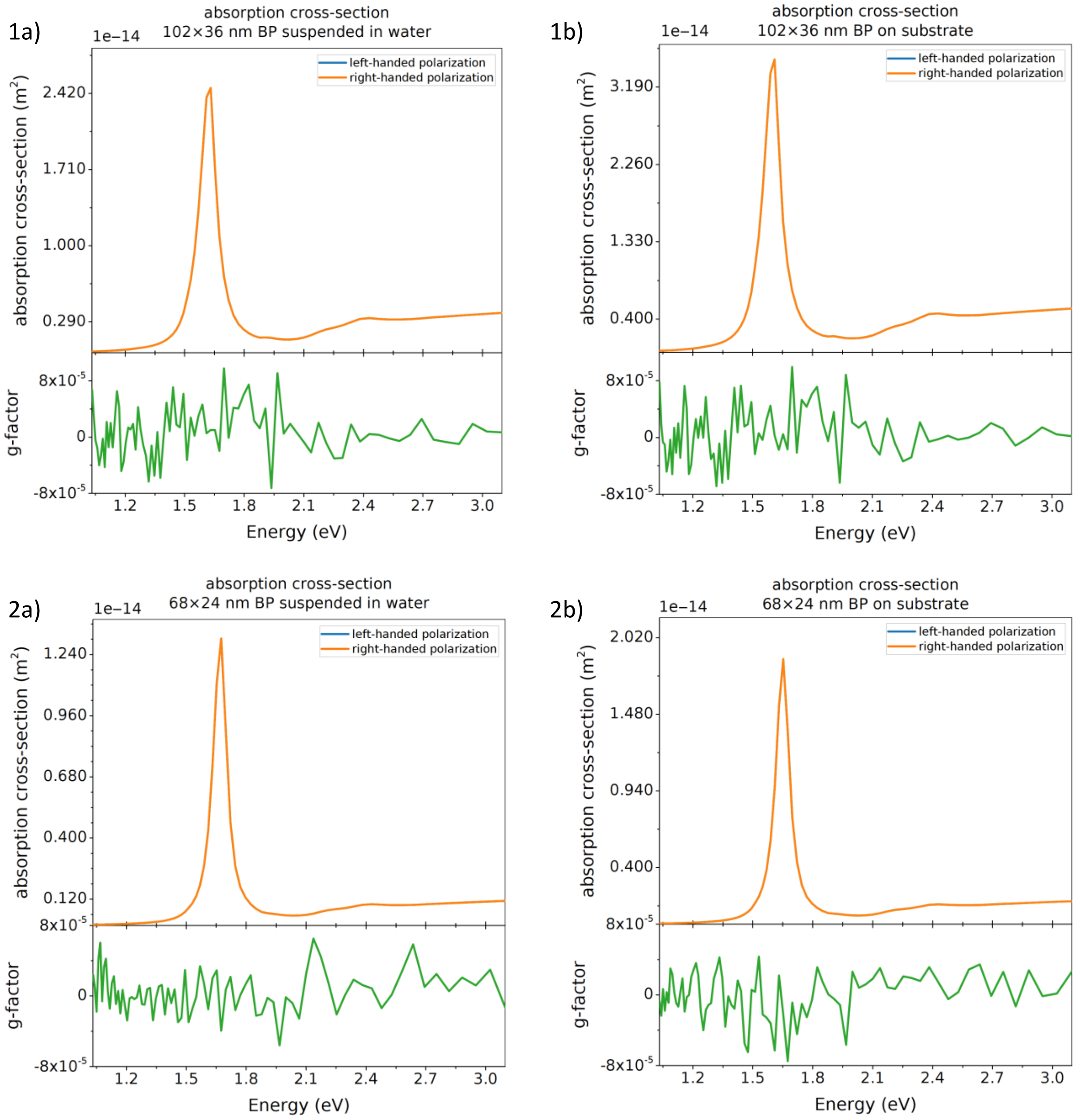

Figure S4 Absorption spectra of 102×36 nm (1a-b) and 68×24 nm (2a-b) single nanobipyramid suspended in water (1-2a) and on substrate (1-2b) with long axis at 31° pitch relative to excitation wavefront. Lorentzian fit in LSPR range of larger particle-on-substrate spectrum yields maximum at 1.597 eV (775 nm) with FWHM of ~0.103 eV (50 nm). Blue line for left-handed polarization in each spectrum is overlapped with orange line for right-handed spectrum. G-factor (defined in the main text) spectra indicate dissymmetry coming merely from numerical noise.

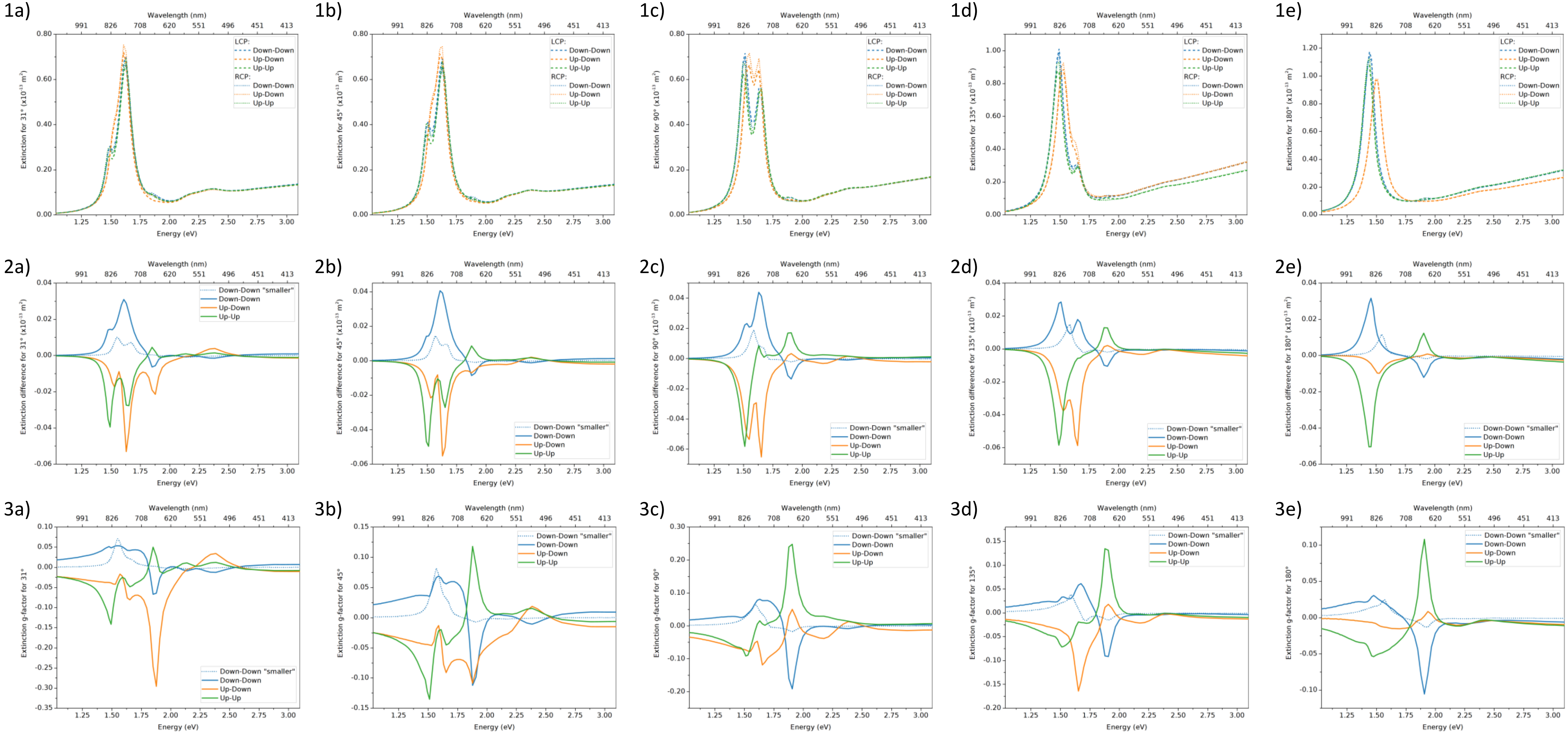

Figure S5 Extinction in 102x36 nm BP dimers as excited by circularly polarized light (1a-e), absolute extinction difference in BP dimers as excited by circularly polarized light (2a-e), and relative extinction difference (g-factor) of BP dimers as excited by circularly polarized light (3a-e). Absolute and relative extinction difference spectra for Down-Down configuration of 68x24 nm BP dimers are presented as dotted lines in rows 2 and 3.

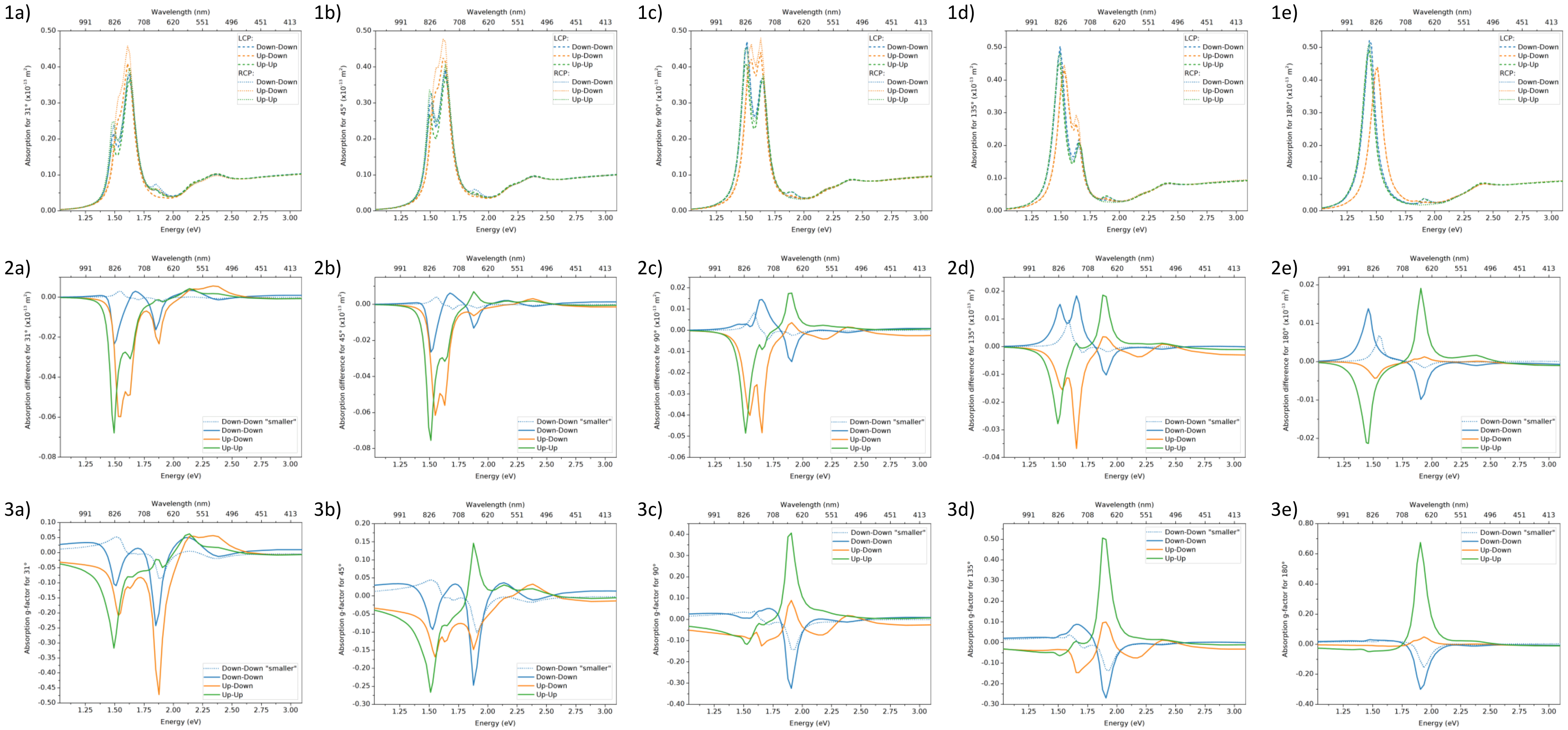

Figure S6 Absorption in 102x36 nm BP dimers as excited by circularly polarized light (1a-e), absolute absorption difference in BP dimers as excited by circularly polarized light (2a-e), and relative absorption difference (g-factor) of BP dimers as excited by circularly polarized light (3a-e). Absolute and relative absorption difference spectra for Down-Down configuration of 68x24 nm BP dimers are presented as dotted lines in rows 2 and 3.

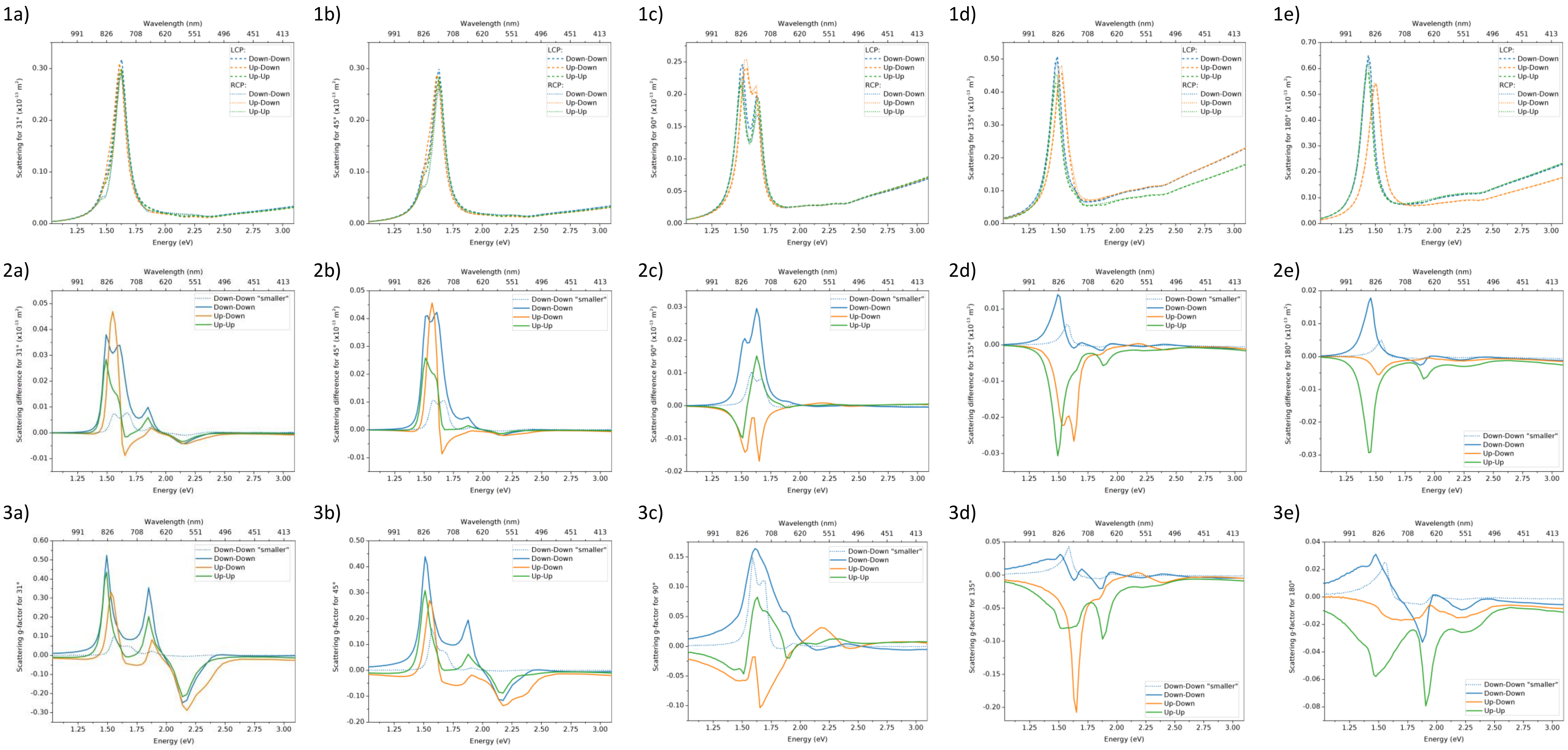

Figure S7 Scattering in 102x36 nm BP dimers as excited by circularly polarized light (1a-e), absolute scattering difference in BP dimers as excited by circularly polarized light (2a-e), and relative scattering difference (g-factor) of BP dimers as excited by circularly polarized light (3a-e). Absolute and relative scattering difference spectra for Down-Down configuration of 68x24 nm BP dimers are presented as dotted lines in rows 2 and 3.

a)

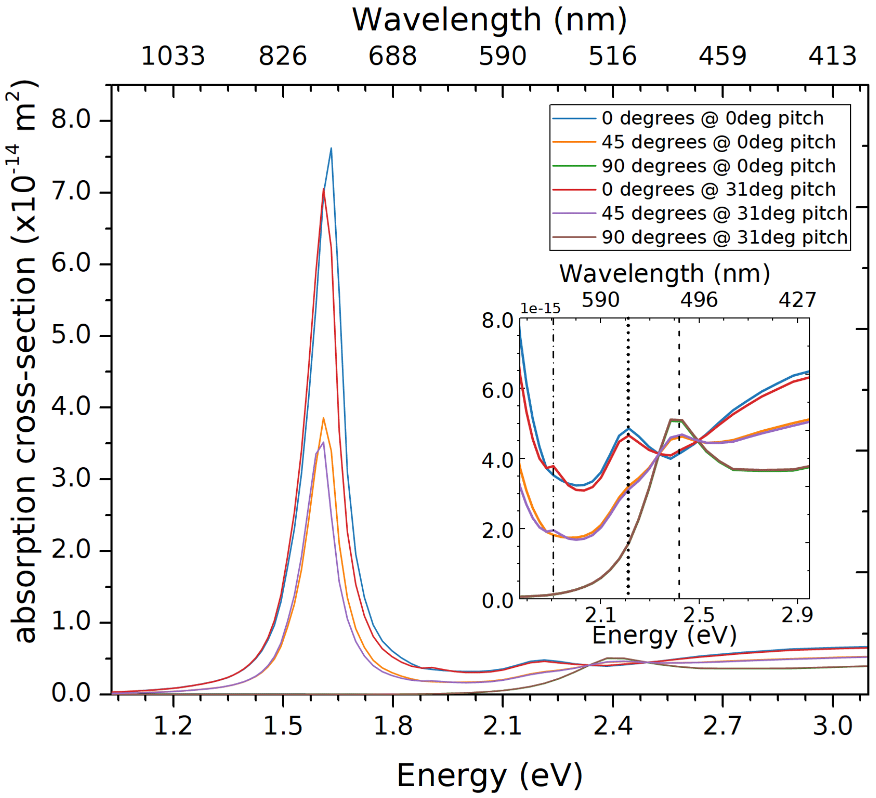

b)

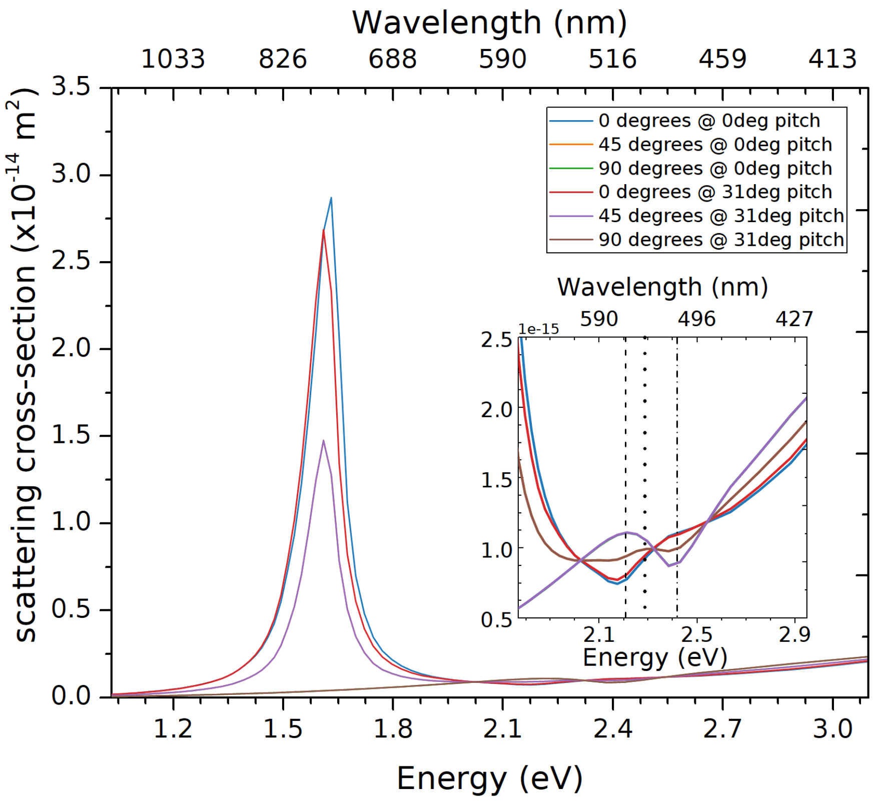

Figure S8 Absorption (a) and scattering (b) cross-sections of single gold bipyramid excited with electromagnetic wave linearly polarized parallel to projection of long (0°) and short (90°) axes of single 102×36 nm BP onto wavefront plane. Wave vector is perpendicular to long axis (0° pitch) and at an angle to long axis (31° pitch resulting from BP lying on one side on substrate). Insets show close-ups on low-amplitude cross-sections in high-energy region.

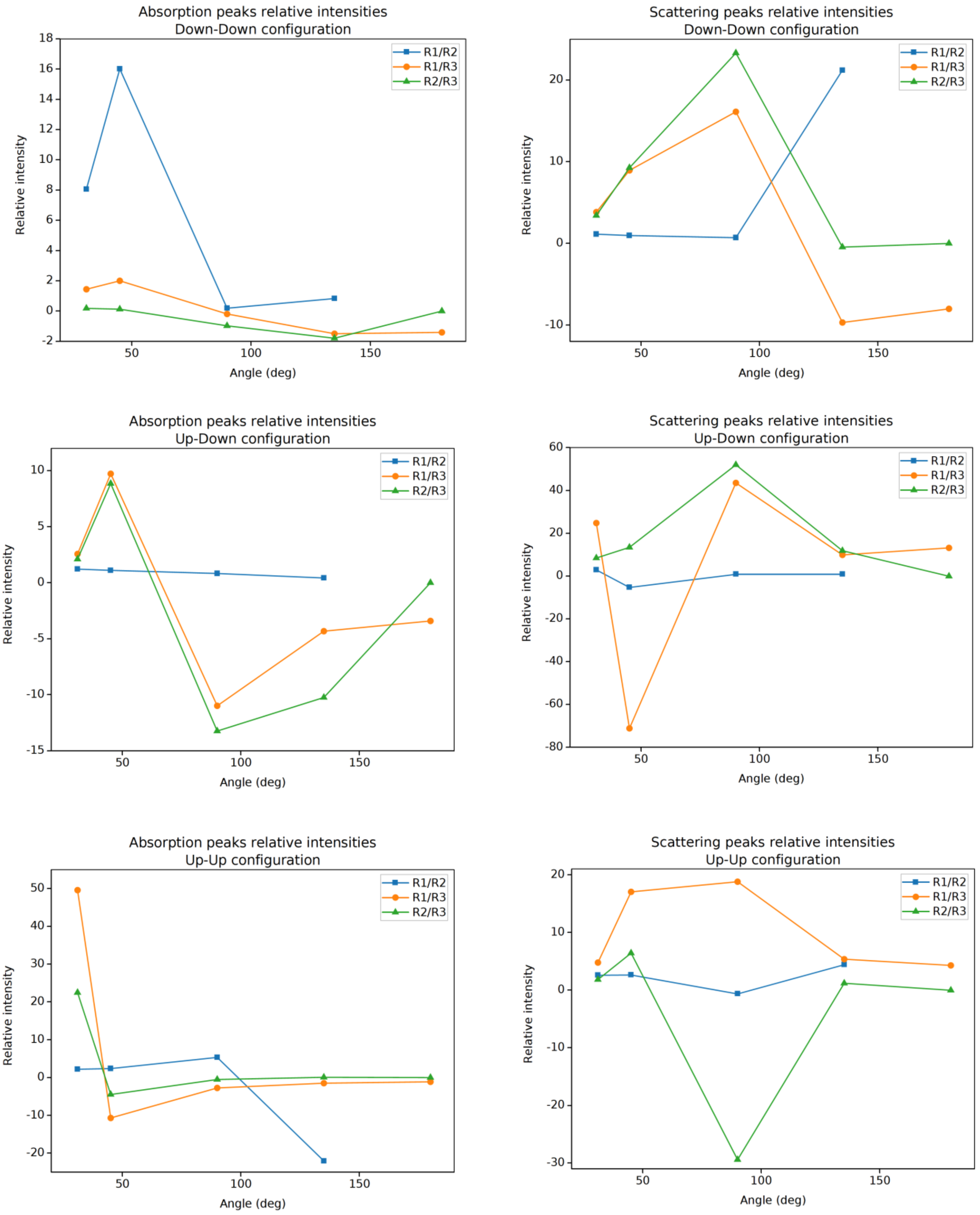

Figure S9 Relative intensities of three lowest-energetic resonances differences for absorption and scattering differential spectra of 102x36 nm BP dimers. R1, R2, and R3 refer to respective resonance amplitude difference at resonances: ~1.5 eV, ~1.65 eV, and ~1.9 eV, respectively. The distance between bipyramids is 5 nm as defined in the main text.

5. References

(1) Bashkatov, A. N.; Genina, E. A., *Water Refractive Index in Dependence on Temperature and Wavelength: A Simple Approximation*; SPIE, 2003; Vol. 5068.

(2) Lide, D. R., *CRC Handbook of Chemistry and Physics, 85th Edition*; Taylor & Francis, 2004.

(3) Schiebener, P.; Straub, J.; Sengers, J. M. H. L.; Gallagher, J. S., Erratum: Refractive Index of Water and Steam as Function of Wavelength, Temperature and Density [J. Phys. Chem. Ref. Data 19, 677 (1990)]. *Journal of Physical and Chemical Reference Data* **1990**, *19*, 1617-1617, DOI: 10.1063/1.555850.

(4) Johnson, P. B.; Christy, R. W., Optical Constants of the Noble Metals. *Physical Review B* **1972**, *6*, 4370-4379, DOI: 10.1103/PhysRevB.6.4370.

(5) Sassaroli, E.; Li, K. C. P.; O'Neill, B. E., Numerical Investigation of Heating of a Gold Nanoparticle and the Surrounding Microenvironment by Nanosecond Laser Pulses for Nanomedicine Applications. *Physics in Medicine and Biology* **2009**, *54*, 5541-5560, DOI: 10.1088/0031-9155/54/18/013.

(6) Yushanov, S.; Crompton, J. S.; Koppenhoefer, K. C., Mie Scattering of Electromagnetic Waves. In *Proceedings of the 2013 COMSOL Conference in Boston*, COMSOL Conference, 2013. URL: https://www.comsol.com/paper/download/181101/crompton_paper.pdf

(7) Sivun, D.; Vidal, C.; Munkhbat, B.; Arnold, N.; Klar, T. A.; Hrelescu, C., Anticorrelation of Photoluminescence from Gold Nanoparticle Dimers with Hot-Spot Intensity. *Nano Letters* **2016**, *16*, 7203-7209, DOI: 10.1021/acs.nanolett.6b03562.

(8) Sánchez-Iglesias, A.; Winckelmans, N.; Altantzis, T.; Bals, S.; Grzelczak, M.; Liz-Marzán, L. M., High-Yield Seeded Growth of Monodisperse Pentatwinned Gold Nanoparticles through Thermally Induced Seed Twinning. *Journal of the American Chemical Society* **2016**, *139*, 107-110, DOI: 10.1021/jacs.6b12143.

(9) Malachosky, E. W.; Guyot-Sionnest, P., Gold Bipyramid Nanoparticle Dimers. *J Phys Chem C* **2014**, *118*, 6405-6412, DOI: 10.1021/jp412409u.
